# Pattern of Malformation in Offspring of Chronic Alcoholic Mothers

**Published:** 1995

**Authors:** Carrie L. Randall, Edward P. Riley

**Affiliations:** Carrie L. Randall, Ph.D., is a professor of psychiatry and director of the Center of Drug and Alcohol Programs at the Medical University of South Carolina, Charleston, South Carolina. Edward P. Riley, Ph.D., is a professor of psychology and director of the Center for Behavioral Teratology, San Diego State University, San Diego, California

**Keywords:** prenatal alcohol exposure, fetal alcohol effects, fetal alcohol syndrome, gestation, congentital anomaly, fetal alcohol development, teratogens

**Figure:**
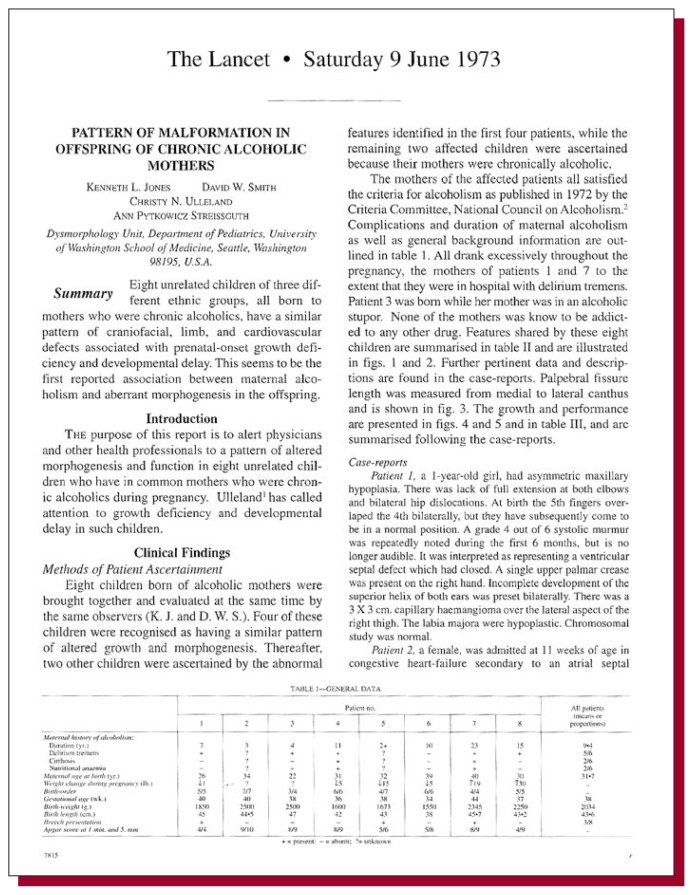
Jones, K.L.; Smith, D.W.; Ulleland, C.N.; and Streissguth, A.P. Pattern of malformation in offspring of chronic alcoholic mothers. *Lancet* 7815:1267–1271, 1973.

The identification of the characteristics defining fetal alcohol syndrome (FAS) is usually credited to observations made in this Jones and colleagues’ 1973 article. Although a previous report in a French medical journal several years earlier recognized a similar pattern (Lemoine 1968), it was Jones and Smith who actually coined the label “fetal alcohol syndrome” in a followup paper to this seminal article by Jones and colleagues, entitled “Recognition of the fetal alcohol syndrome in early infancy” (Jones and Smith 1973).

Historically, it was considered common knowledge that alcohol consumption during pregnancy was hazardous, and women were warned accordingly; around the turn of the century, there were even a few clinical and experimental reports published that outlined negative effects of such consumption. However, the clinical inquisitiveness and astute diagnostic skills of Jones and the rest of the team of physicians at the University of Washington, led by the late Dr. David W. Smith, was the impetus for the work published in this landmark article.

The observed characteristics caused by maternal drinking and reported by Jones and colleagues in the article interested practicing clinicians around the world, stimulating many international case reports that further confirmed these findings. The observations in the article also had a monumental impact on the scientific community. They demonstrated that alcohol ingested by the mother adversely affected fetal growth and development. Thus, as with thalidomide, alcohol was being implicated as a teratogen, or an agent capable of altering normal fetal growth and development, and this was an exciting development.

Since the original identification of FAS in 1973, approximately 600 additional cases have been reported in the alcohol literature. Abel (1990) published a compendium of these cases. Basically, all the cardinal features of FAS described in the 1973 article by Jones and colleagues of low birth weight; small eye openings; joint, limb, and cardiac defects; developmental delays; and mental retardation have withstood the test of time. In addition, some anomalies not initially included in the characteristics they outlined, such as a thin upper lip (narrow vermilion border) and a broad flat area under the nose (long smooth philtrum), are now considered features of FAS.

One area of science inadvertently stimulated by this article was that of behavioral teratology. In an attempt to develop animal models of FAS, researchers found that prenatal alcohol exposure in rats was associated with behavioral anomalies in the absence of gross physical defects. This meant that the brain could be affected while other major organ systems appeared normal. As a result of this research, alcohol today is considered by many scientists as a substance that leads to birth defects manifested in behavioral problems.

Jones and colleagues’ work also has had a tremendous impact on society in general. In 1989, warning labels issued by the U.S. Surgeon General began appearing on alcoholic beverage containers, and they included the phrase “. . . women should not drink alcoholic beverages during pregnancy because of the risk of birth defects.” Numerous States have statutes concerning the issue of drinking during pregnancy and/or have established a department or agency to deal with issues related to prenatal alcohol exposure. A national newsletter, the *Iceberg*, published in Seattle, WA, helps parents raising children with FAS, and a national organization, the National Association on Fetal Alcohol Syndrome, has been established to increase awareness of the adverse consequences of drinking during pregnancy to help prevent FAS.

A search of the National Institute on Alcohol Abuse and Alcoholism’s Alcohol and Alcohol Problems Science Database using “fetal alcohol syndrome” or “fetal alcohol effect” as key words revealed that approximately 2,800 articles appeared between 1973 and 1993 attempting to substantiate, clarify, and understand the various ramifications of prenatal alcohol exposure in both humans and animals. Additionally, in the last decade, investigators have published articles that have attempted to identify potential mechanisms of action of alcohol on the body from the physiological to the molecular level.

Further evidence of this article’s importance to the alcohol field is the fact that this 20-year old article has been cited more than 1,050 times (*Science Citation and Social Science Citation Index* 1994). To put this in perspective, the median citation count for articles published in 12 alcohol and drug journals in 1984 was two citations over a 4-year period (Howard and Howard 1992). The more than 2,300 basic and clinical research papers confirming the teratogenicity of alcohol in many different species can be attributed to Jones and colleagues’ seminal article.

No doubt this initial observation has had far-reaching clinical, scientific, societal, and political implications of greater significance than the authors could have imagined.
